# Internet addiction among nursing students: application of latent profile analysis and network analysis

**DOI:** 10.3389/fpsyt.2026.1814343

**Published:** 2026-04-21

**Authors:** Ying Li, Meng Xiang, Jinyuan Su, Jing Hu

**Affiliations:** 1 College of Sports Science, Jishou University, Jishou, Hunan, China; 2 School of Physical Education and Arts, Hunan University of Medicine, Huaihua, Hunan, China; 3 College of Physical Education and Health, Zhangjiajie College, Zhangjiajie, Hunan, China; 4 Operating Room, Nanjing Gaochun People's Hospital, Nanjing, Jiangsu, China

**Keywords:** internet addiction, LPA, mental health, network analysis, nursing education

## Abstract

**Background:**

Internet addiction is widely reported and heterogeneous among nursing students. However, variable-centered approaches may not fully capture profile differences and core symptom patterns, potentially limiting precise interventions. Therefore, identifying distinct profiles and key symptoms is important for informing effective prevention.

**Objective:**

This study aims to identify distinct internet addiction profiles among nursing students, explore the characteristics and core symptoms of these profiles, and investigate the factors associated with their variation.

**Methods:**

A cross-sectional survey was conducted among undergraduate nursing students from September to November 2025. Latent profile analysis (LPA) and network analysis were performed to characterize the patterns of problematic internet use across identified profiles.

**Result:**

Latent Profile Analysis revealed four distinct problematic internet use profiles: No-Problematic Internet Use Profile (17.895%), Low-Problematic Internet Use Profile (41.957%), Moderate-Problematic Internet Use Profile (26.676%), and High-Problematic Internet Use Profile (13.472%). Multinomial logistic regression identified gender, monthly household income, and physical activity as significant factors associated with profile membership. Network analysis highlighted central symptoms specific to each profile: Health-related problems (RP-IH) and compulsive internet use and withdrawal symptoms (Sym-C & Sym-W) exhibited the highest centrality within the Moderate- and High-Problematic Internet Use Profiles.

**Conclusion:**

Internet addiction among undergraduate nursing students is a heterogeneous phenomenon that can be categorized into four distinct profiles. Our findings clarify key associated factors and identify central symptoms specific to each profile, potentially providing an empirical basis for nursing educators to develop targeted psychological interventions.

## Introduction

The widespread integration of digital technology into daily life has fundamentally reshaped behavioral patterns and social interactions in contemporary society (1). While digital connectivity supports academic and social needs, excessive and maladaptive internet use has emerged as a significant public health concern, particularly among adolescents and young adults (2). Internet Addiction was defined by the American psychologist Young (3) as an impulse control disorder involving impulsive behavior and dependence on internet use without the involvement of addictive substances, which ultimately compromises social functioning. According to the meta-analysis by Yiman Liu (2021), the detection rate of internet addiction among college students ranged between 10.2% and 13.2% during the period 2011–2018 (4). Among high-stress student populations, such as medical students, this prevalence rises to 29% (5, 6). These findings clearly indicate that internet addiction has become a prominent and persistent challenge among university students in China. Internet addiction is often accompanied by a range of psychological and behavioral issues, such as mood disorders (7, 8), compromised physical health (9), sleep disturbances (10), academic underachievement (11), and suicidal behaviors (12). For nursing students, the occurrence of internet addiction poses a serious threat to educational success and career development. It disrupts engagement with high-intensity curricula and adversely affects academic performance (13). In clinical settings, internet addiction undermines clinical reasoning and decision-making in patient care, impairs the development of professional identity, and erodes the emotional resilience essential to nursing practice (14).

Traditional studies have predominantly assessed the severity of Internet Addiction across populations based on total scale scores (15, 16). However, empirical evidence suggests that internet addiction is not merely a binary construct (“present” vs. “absent”); individuals classified as having internet addiction may exhibit qualitative differences (17). This methodological limitation has led to inefficient and imprecise management of internet addiction within this population. Latent Profile Analysis (LPA) is a probability model based on observed variables that classifies individuals into homogeneous latent profiles, thereby identifying distinct groups with similar response patterns (18). This method facilitates the identification of distinct characteristics among profiles, potentially informing the development of targeted interventions (19). For instance, Yang et al. (2020) utilized LPA to identify three latent classes of internet addiction among college students: “High-risk” (21.6%), “Intermediate” (53.0%), and “Low-risk” (25.4%) (20). Further research indicates that individuals with varying levels of internet addiction differ not only in symptom severity but also in underlying psychological mechanisms and the extent of functional impairment (21, 22). Although existing literature has explored the heterogeneity of internet addiction among general college students, studies focusing specifically on nursing students remain scarce. Therefore, by identifying profiles of internet addiction among nursing students, this study provides a foundation for tailored care for different profiles, which may enhance the effectiveness of future interventions.

Furthermore, clearly defining the core dimension is crucial, as it serves as the primary focus for intervention strategies. Given the multiple dimensions of internet addiction, as outlined in the symptom science literature, prioritizing this core dimension helps concentrate resources and enhance efficiency. Therefore, conducting a thorough analysis of the internal structure of social isolation is essential. Only through such in-depth investigation can we accurately identify the core dimension of internet addiction, thereby enabling the formulation of targeted interventions. As an innovative research method, network analysis (NA) employs visualization and quantitative approaches to capture the complex interrelationships among various dimensions of internet addiction, providing a powerful tool for a deeper understanding of its internal structural characteristics (23). NA theory, nodes with high centrality indices can serve as key targets for therapeutic interventions due to their significant influence within the network (24). Interventions targeting these critical nodes are likely to produce cascading effects, thereby alleviating issues in other dimensions. Moreover, incorporating factors influencing internet addiction into NA not only helps reveal underlying mechanisms but also establishes a solid theoretical foundation for developing more effective and precise intervention strategies, ultimately facilitating the efficient implementation and ongoing optimization of these interventions (24).

Based on the aforementioned research, this study aims to achieve three primary objectives: first, to identify distinct latent profiles of problematic internet use among undergraduate nursing students using LPA; second, to clarify the factors associated with profile membership; and third, to apply NA to examine the core characteristics of problematic internet use within each identified profile. The findings are expected to provide valuable insights for developing targeted psychological interventions tailored to the specific needs of each profile within this population.

## Materials and methods

### Participants

This study recruited nursing students from seven universities across five provinces (Shandong, Hubei, Hunan, Henan, and Jiangxi) between September and November 2025 using a convenience sampling approach. Data were collected through anonymous self-administered questionnaires distributed by class advisors. Questionnaires were administered continuously until no new responses were received for seven consecutive days, at which point data collection ceased. Among the 1,563 completed questionnaires, 153 were excluded due to missing items or uniform responses, resulting in 1,492 valid responses retained for analysis.

The inclusion criteria were as follows: (1) enrollment in an undergraduate nursing degree program; (2) sufficient proficiency in Mandarin to ensure smooth communication; and (3) willingness to provide informed consent.

The exclusion criteria included: (1) Participants with documented mental disorder diagnoses in medical records were excluded to reduce confounding from comorbid psychopathology; and (2) being on sick leave or academic suspension during the study period.

### Measures

#### Social demographic information

Social demographic information includes gender (male or female), age(≤20 (25), 21-23and ≥24), living status (urban or rural), educational level, monthly household income and family atmosphere (participants’ perceived family decision-making style).

#### Physical activity rating scale

The Physical Activity Rating Scale-3 (PARS-3), originally developed by Liang Deqing (1994) (26), was used to evaluate participants’ physical activity levels over the preceding week. This instrument assesses physical activity across three domains: intensity, frequency, and duration, each rated on a 5-point scale. Intensity and frequency scores range from 1 to 5 points, while duration is scored from 0 to 4 points. A composite physical activity score (range: 0–100) is derived by multiplying the scores of the three dimensions. Higher total scores indicate more vigorous physical activity levels. In this study, the Cronbach’s α for the sample was 0.830.

#### Internet addiction

The Chen Internet Addiction Scale (CIAS-R) was originally developed by Professor Shuhui Chen in Taiwan in 1999 (27), with a sample of college students. In this study, the revised version by Bai Yu et al (28). was adopted as the measurement tool. This scale comprises four dimensions: compulsive symptoms, tolerance symptoms, interpersonal and health problems, and time management issues, with a total of 19 items. A Likert 4-point scoring method (1 = “strongly disagree” to 4 = “strongly agree”) was employed. Scores < 46 were classified as healthy, scores 46–53 as internet dependence, and scores > 53 as internet addiction (28). In this study, the Cronbach’s α for the sample was 0.918.

### Statistical analysis

Data analysis was conducted using Mplus 8.3, SPSS 26.0 (IBM, Armonk, NY, USA), and the R statistical software (version 4.3.1). Descriptive statistics were computed for all variables. Continuous variables are described as mean and standard deviation (SD), while categorical variables are summarized using frequencies and percentages.

LPA was employed to identify distinct profiles of problematic internet use among undergraduate nursing students, based on item-level responses to the Internet Addiction Test. Model fit was assessed using the Akaike Information Criterion (AIC), Bayesian Information Criterion (BIC), and sample-size adjusted BIC (aBIC), with lower values indicating superior fit. Classification accuracy was evaluated by entropy, where values closer to 1 are preferred (29). The optimal number of classes was determined by comparing the k-class model against the k-1 class model using the Bootstrapped Likelihood Ratio Test (BLRT) and the Lo-Mendell-Rubin Likelihood Ratio Test (LMRT); a significant p-value for these tests suggests that the k-class model provides a better fit (30).

The chi-square test was used to compare categorical variables across profiles. For continuous variables that did not meet the assumption of normality, the Kruskal-Wallis H test was applied to assess differences among profiles. Where significant omnibus effects were detected, *post-hoc* pairwise comparisons were conducted using the Bonferroni correction. Variables that demonstrated statistically significant differences in the univariate analyses were subsequently entered into a multinomial logistic regression.

Network analysis was conducted using the R packages qgraph and bootnet. A Gaussian Graphical Model (GGM) was estimated to visualize the relationships among variables. Edge weights were regularized using the graphical Least Absolute Shrinkage and Selection Operator (gLASSO), and the Extended Bayesian Information Criterion (EBIC) was applied for model selection with a tuning parameter of γ = 0.5. For centrality indices, we focused on Strength, which measures the sum of absolute edge weights connected to each node and reflects the node’s overall influence within the network. Network stability for both edge weights and centrality indices was assessed using nonparametric bootstrap analysis with 1,000 replications. The correlation stability (CS) coefficient was calculated, with CS > 0.25 indicating acceptable stability and CS > 0.50 indicating good stability (31).

## Results

### Participant characteristics

A total of 1492 nursing students were enrolled in this study. 1185 participants (79.424%) were female and 307(20.576%) were male. Detailed characteristics of the participants are presented in **Table 1**.

**Table 1:** Demographic and academic-related characteristics by latent profiles (N = 1492).

Variable	No problematic internet use profile (267)	Low problematic internet use profile (626)	Moderate problematic internet use profile (398)	High problematic internet use profile (201)	χ²/Z	*P*
Gender					16.029	0.001
Male	67	117	66	57		
Female	200	509	332	144		
Age					80.804	<0.001
<20 Year	70	269	238	106		
21–23 Year	168	308	145	82		
≥24 Year	29	49	15	13		
Only Child (OC)					12.114	0.007
Yes	77	136	82	32		
No	190	490	316	169		
Family Residence					10.573	0.014
Urban	152	294	185	88		
Rural	115	332	213	113		
Monthly Household Income(¥)					41.736	<0.001
≤3000	33	87	30	41		
3001-6000	84	245	159	88		
6001-9000	72	175	123	39		
≥9001	78	119	86	33		
Clinical Practice (CP)					61.417	<0.001
Yes	115	188	73	35		
No	152	438	325	166		
Family Atmosphere					64.755	<0.001
Highly Democratic	141	243	106	53		
Moderately Democratic	112	352	260	125		
Authoritarian	14	31	32	23		
Nursing Interest (NI)					69.659	<0.001
Dislike	22	48	30	32		
Neutral	164	460	330	148		
Like Very Much	81	118	38	21		
Physical Activity (PA)	20.715 ± 18.633	17.255 ± 16.612	15.704 ± 15.037	14.910 ± 17.669	6.234	<0.001

### Latent profile analysis of internet addiction

**Figure 1:**
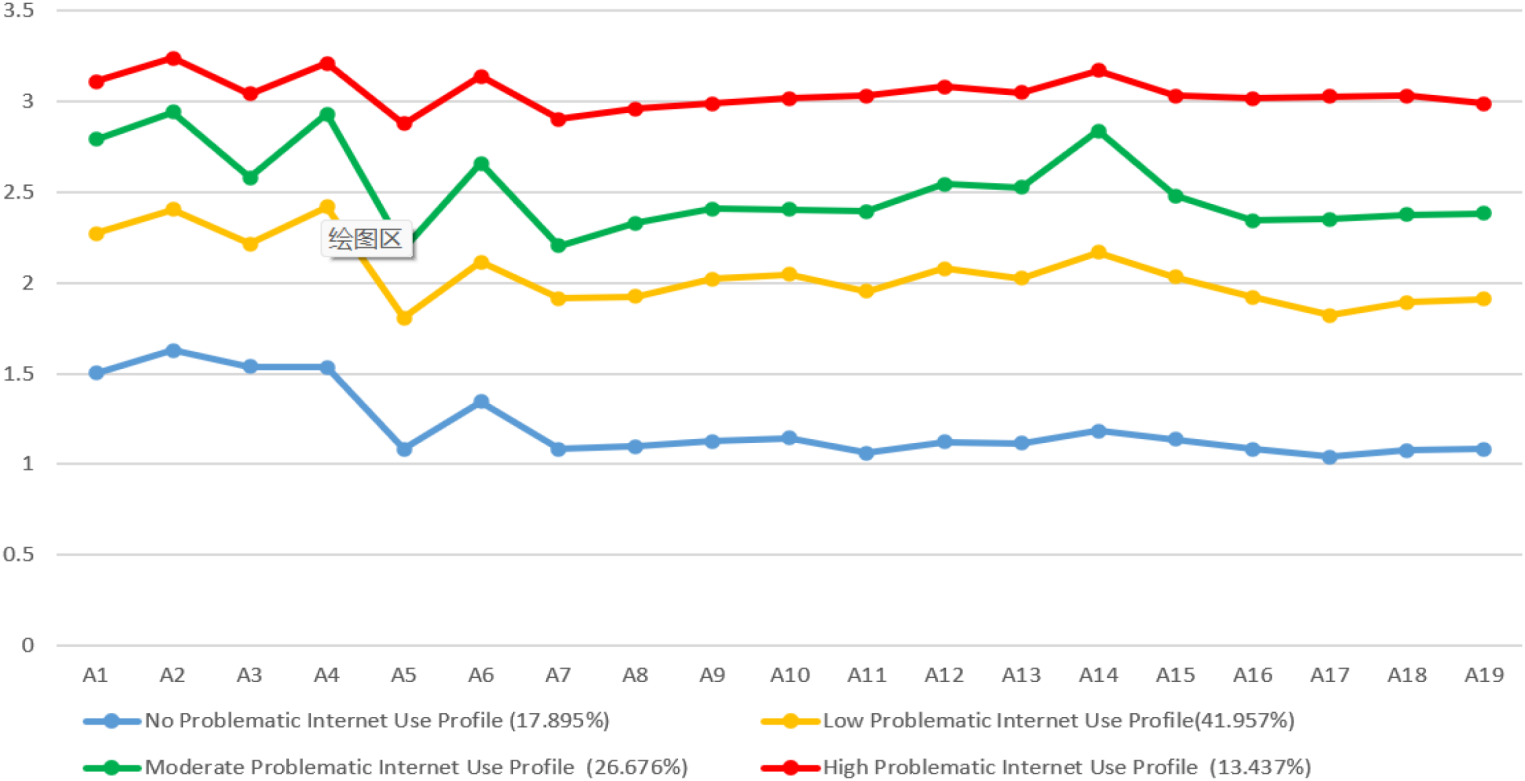
Estimated sample item means for the three latent profiles of internet addiction.

Using the items of the Internet Addiction Test as observed indicators, this study employed LPA to construct models ranging from 1 to 5 profiles to explore the latent profile structure of Problematic Internet Use among undergraduate nursing students (**Table 2**). As the number of profiles increased from 1 to 5, the values of AIC, BIC, and aBIC consistently decreased, reaching their minimum values in the 4-profile model compared to the 1- to 3-profile models. The 5-profile model was rejected because the Lo-Mendell-Rubin adjusted Likelihood Ratio Test (LMRT) was not statistically significant (p > 0.05). Although the 4-profile model had the lowest Entropy value among the candidate models, it still exceeded 0.90, indicating good classification accuracy. Moreover, the 4-profile solution offered a clearer and more interpretable characterization of the distinct patterns of internet addiction among nursing students. Therefore, the 4-profile model was determined to be optimal. The four latent profiles exhibited distinct conditional probability scores across all internet addiction items, showing a progressive pattern. Based on score characteristics and profile proportions, they were labeled as: C1 “No Problematic Internet Use Profile “ (17.895%), with scores approaching the minimum; C2 “Low Problematic Internet Use Profile” (41.957%), slightly above the midpoint; C3 “Moderate Problematic Internet Use Profile “ (26.676%), moderately elevated; and C4 “High Problematic Internet Use Profile “ (13.437%), approaching the maximum, see **Figure 1**.

**Table 2:** Model fit indices for latent profile analysis.

Indices	Unconditional model				
	1-profile	2-profile	3-profile	4-profile	5-profile
Fit statistics					
AIC	65690.623	55031.413	49198.940	47833.854	46905.475
BIC	65892.322	55339.269	49612.954	48354.026	47531.804
aBIC	65771.607	55155.020	49365.170	48042.708	47156.951
Entropy	–	0.982	0.955	0.931	0.930
BLRT	–	<0.001	<0.001	<0.001	0.473
LMRT	–	<0.001	<0.001	<0.001	0.476
Group sizes (%)					
C1	100%	22.520%	18.767%	17.895%	13.606%
C2		77.480%	55.563%	41.957%	7.507%
C3			25.670%	26.676%	26.810%
C4				13.472%	38.472%
					13.606%

LL, Log-likelihood; AIC, Akaike Information Criterion; BIC, Bayesian Information Criterion; aBIC, adjusted BIC; LMR, Lo–Mendell–Rubin likelihood ratio test.

### Demographic and academic-related characteristics of each profile

The results of univariate analysis showed that there were significant differences among the three potential characteristics of gender, age, only child (OC), monthly family income, clinical practice (CP), family atmosphere, nursing interest (NI) and physical activity (PA) (P < 0.05). The results are shown in **Table 1**.

### Predictor of latent profile membership

Using the “ No Problematic Internet Use Profile “ group as the reference, multivariate logistic regression was performed to identify the predictive factors (**Table 3**). Nursing students in the younger age group (<20 years) showed a greater tendency to belong to the “Moderate Problematic Internet Use Profile “ group. Female gender, strong interest in the nursing profession (“Like Very Much”), and a highly democratic family atmosphere were identified as protective factors in the “High Problematic Internet Use Profile “ group. Details are presented in **Table 3**.

**Table 3:** Multiple logistic regression analysis of influencing factors of internet addiction among undergraduate nursing students.

Variables	Low problematic internet use profile vs no problematic internet use profile				Moderate problematic internet use profile vs no problematic internet use profile				High problematic internet use profile vs no problematic internet use profile			
	β	SE	OR	*P*	β	SE	OR	*P*	β	SE	OR	*P*
Gender (reference: male)												
Female	0.080	0.196	1.083	0.683	0.007	0.225	0.974	0.001	-0.806	0.243	0.446	<0.001
Age (reference:≥24 year)												
<20 Year	0.642	0.317	4.098	0.043	1.410	0.400	4.908	<0.001	0.719	0.437	2.051	0.100
20–23 Year	-0.083	0.270	0.094	0.760	0.152	0.361	1.164	0.673	-0.229	0.395	0.795	0.561
Only child (OC) (No)												
Yes	-0.123	0.184	0.442	0.506	-0.039	0.210	0.035	0.852	-0.479	0.261	3.360	0.067
NI (reference: dislike)												
Like or Very Much	-0.158	0.313	0.854	0.614	-0.615	0.370	0.540	0.096	-1.314	0.398	0.269	<0.001
Neutral	0.259	0.285	1.296	0.362	0.404	0.316	1.497	0.202	-0.469	0.320	0.625	0.143
Family atmosphere (reference: authoritarian)												
highly Democratic	-0.220	0.366	0.803	0.548	-1.039	0.381	0.354	0.006	-1.218	0.412	0.296	0.003
Moderately Democratic	0.211	0.364	1.235	0.562	-0.179	0.373	0.836	0.631	-0.400	0.397	0.670	0.314
Family residence (reference: Rural)												
Urban	-0.204	0.168	0.815	0.223	-0.305	0.187	0.737	0.103	-0.120	0.217	0.887	0.580
Monthly household Income(¥ reference: >9000)												
<3000	0.422	0.270	1.525	0.118	-0.439	0.327	0.645	0.179	0.783	0.345	2.189	0.023
30001-6000	0.448	0.207	1.564	0.031	0.186	0.231	1.205	0.420	0.602	0.281	1.826	0.032
6001-9000	0.373	0.212	1.452	0.078	0.218	0.234	1.244	0.351	0.084	0.303	1.087	0.782
Clinical practice (reference: No)												
Yes	-0.244	0.181	0.784	0.179	-0.514	0.217	0.598	0.018	-0.856	0.268	0.425	0.001
Physical activity (reference: High)												
Low physical activity					1.907	0.294	6.731	<0.001				
Moderate physical activity					1.361	0.322	3.899	<0.001				

### Network analysis across latent profiles

**Figure 2:**
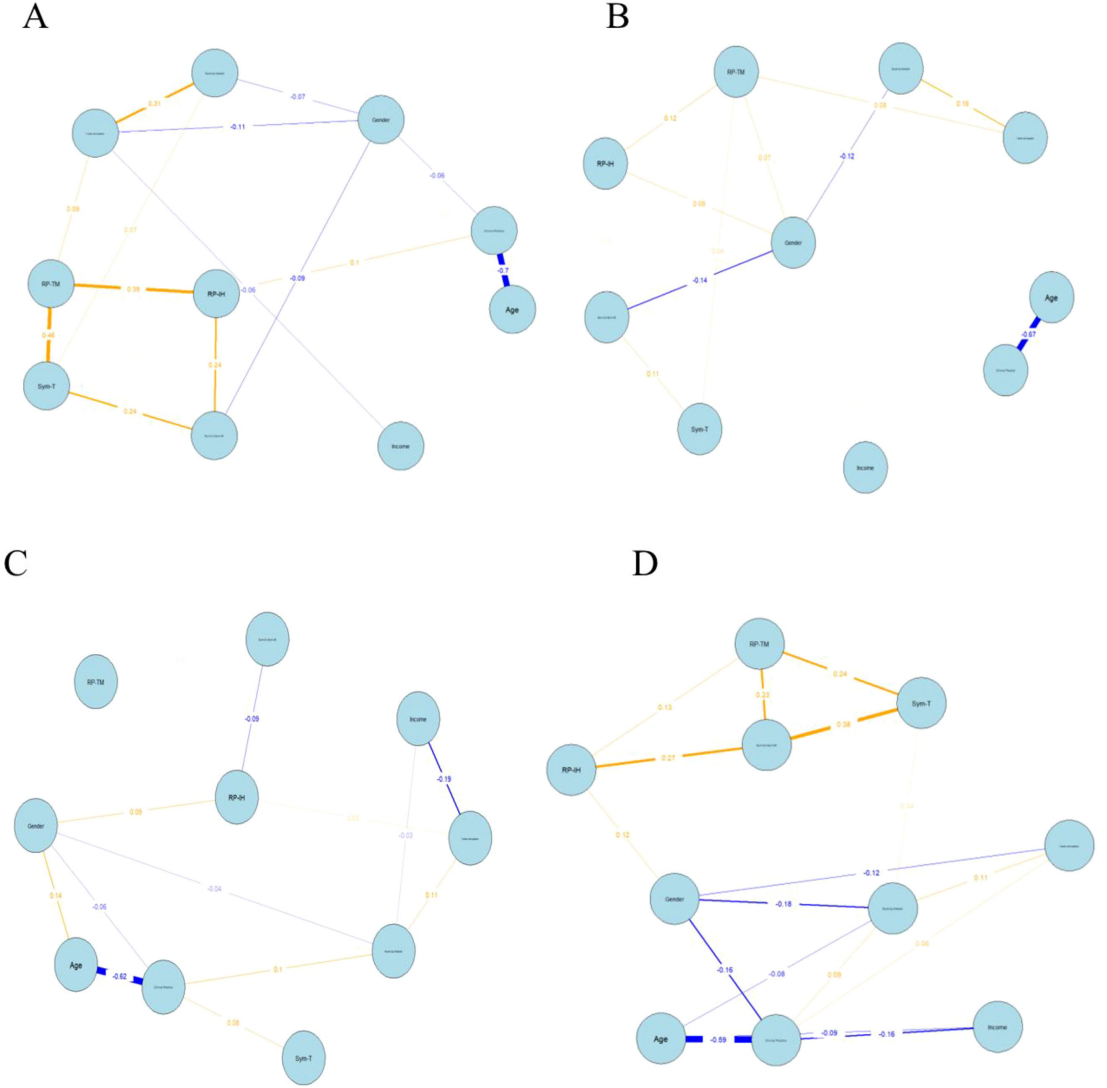
The networks of the four latent profiles. **(A)** No Problematic Internet Use Profile **(B)** Low Problematic Internet Use Profile; **(C)** Moderate Problematic Internet Use Profile; **(D)** High Problematic Internet Use Profile. Interpersonal and Health Problems (RP-IH); Time Management Problems (RP-TM); Internet Addiction Withdrawal Symptoms (Sym C & Sym-W) and Tolerance of Internet Addiction (Sym-T). Yellow indicates a positive correlation, while blue indicates a negative correlation. The thickness of the edge reflects the magnitude of the correlation.

**Figure 3:**
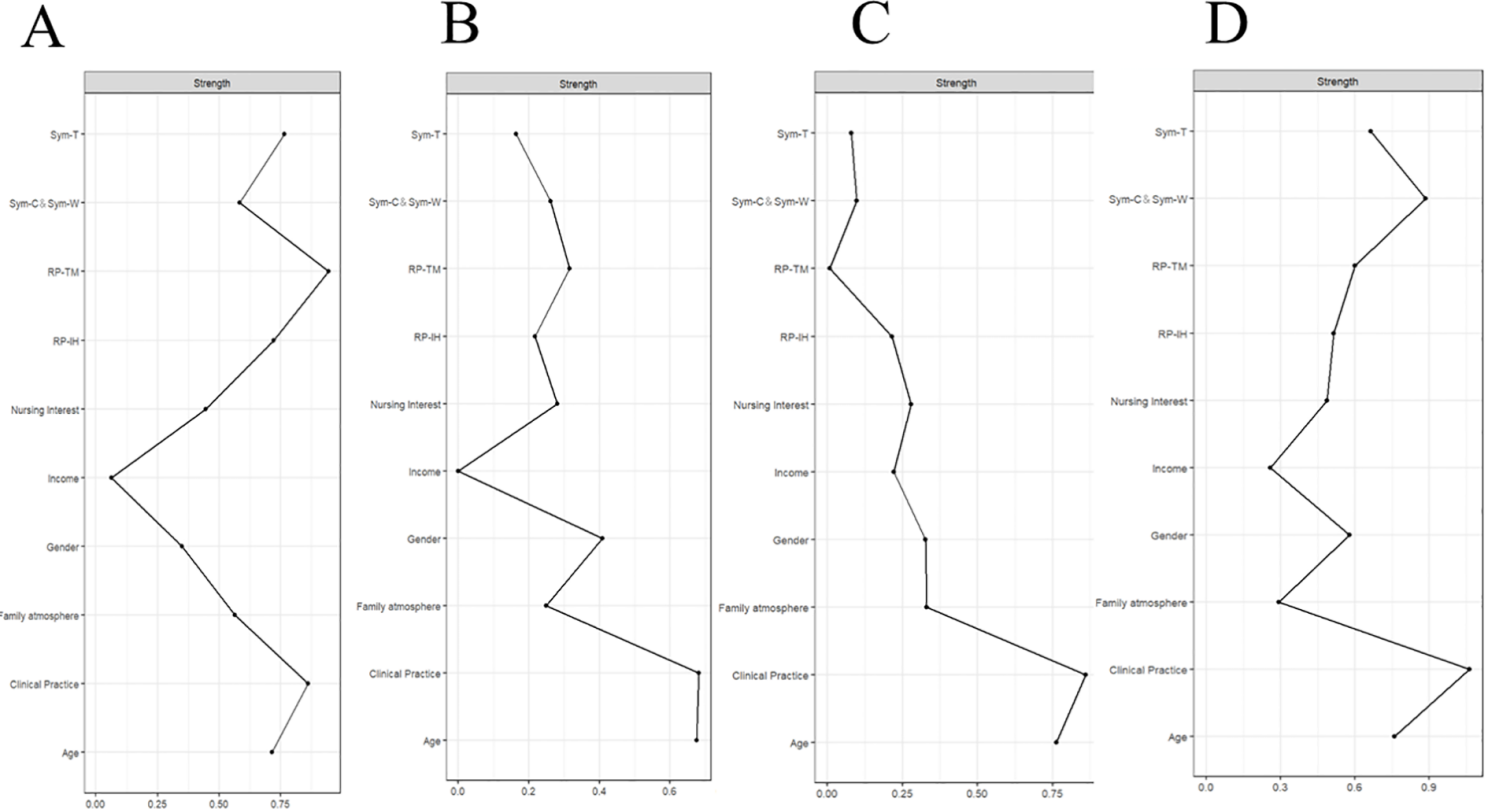
The strength centrality index of the networks of the three latent profiles. **(A)** No Problematic Internet Use Profile **(B)** Low Problematic Internet Use Profile; **(C)** Moderate Problematic Internet Use Profile; **(D)** High Problematic Internet Use Profile.

The network graphs and centrality indices for the three dimensions of internet addiction and their influencing factors are presented in **Figures 2A–D**, **3A–D** and **Supplementary Material**, respectively. The network structures of the Low-, Moderate-, and High-Problematic Internet Use Profiles are illustrated in **Figures 2B–D**. In the low problematic internet use profile (**Figure 2B**), significant positive correlations were observed between Gender and Interpersonal and Health Problems (RP-IH) (coefficient = 0.08), between Gender and Time Management Problems (RP-TM) (coefficient = 0.07), and between RP-IH and RP-TM (coefficient = 0.12). In the moderate problematic internet use profile (**Figure 2C**), significant positive correlations were identified between Gender and RP-IH (coefficient = 0.09), and between Clinical Practice and Tolerance of Internet Addiction (Sym-T) (coefficient = 0.08). In the high problematic internet use profile (**Figure 2D**), stronger associations emerged, with significant positive correlations between Compulsive Internet Use and Withdrawal Symptoms (Sym-C & Sym-W) and Sym-T (coefficient = 0.38), and between RP-IH and Sym-C & Sym-W (coefficient = 0.27). These findings indicate that as internet addiction severity increases, the network shifts from demographic and behavioral factors (e.g., Gender, Clinical Practice) toward core addiction symptoms (e.g., tolerance, withdrawal, compulsive use).

Strength centrality analysis indicated variations in node connectivity across the three profiles (**Figures 3B–D**). In the low problematic internet use profile (**Figure 3B**), RP-TM exhibited the highest strength centrality (strength = 0.3145), indicating that time management problems were the most centrally connected node within this network. In the moderate problematic internet use profile (**Figure 3C**), RP-IH (strength = 0.214) and Clinical Practice (strength = 0.861) showed relatively higher connectivity, suggesting a pattern where interpersonal and health-related problems are more strongly associated with other symptoms in this profile compared to the low-risk profile. In the high problematic internet use profile (**Figure 3D**), Sym-C & Sym-W (strength = 0.885) and Clinical Practice (strength = 1.063) remained the most central nodes. The correlation stability coefficients ranged from 0.517 to 0.751 across the four problematic internet use profiles (No-Problematic Internet Use Profile: 0.670; Low-Problematic Internet Use Profile: 0.751; Moderate-Problematic Internet Use Profile: 0.595; High-Problematic Internet Use Profile: 0.517). Bootstrap 95% confidence intervals for all edge weights are reported in **Supplementary Material**.

## Discussion

This study employed Latent Profile Analysis (LPA) to explore the heterogeneity of problematic internet use among undergraduate nursing students. Four distinct profiles were identified: No-Problematic Internet Use Profile (17.9%), Low-Problematic Internet Use Profile (42.0%), Moderate-Problematic Internet Use Profile (26.7%), and High-Problematic Internet Use Profile (13.5%). Additionally, network analysis was conducted across these profiles to examine symptom connectivity. Our findings reveal that factors associated with profile membership vary among nursing students across different profiles. Further analysis demonstrates that central symptoms differ across profiles, suggesting distinct connectivity patterns within each network. The results highlight the potential value of developing personalized intervention strategies tailored to specific profiles within nursing education programs. These findings offer insights into symptom-level patterns and provide evidence-based implications for informing nursing education practices.

In the low, moderate, and high problematic internet use profiles, progressively higher scores were observed for items 2, 4, 6, and 14. These items primarily belong to the dimension of Tolerance of Internet Addiction (Sym-T)—defined as the phenomenon wherein, with prolonged internet use, individuals require progressively more online content or extended engagement duration to attain a level of satisfaction equivalent to that initially achieved (32). Excessive and persistent internet use continuously stimulates the brain’s reward system, leading to adaptive downregulation of dopamine D2 receptors (DRD2) and consequently reduced receptor responsiveness (33). Neuroimaging evidence indicates that individuals with internet addiction disorder (IAD) exhibit significantly decreased DRD2 availability and dopamine transporter (DAT) levels in the striatal region, suggesting concurrent impairments in dopamine signaling and reuptake processes (34). These dual abnormalities collectively disrupt reward circuit function, thereby driving an increased demand for internet-related stimulation and further exacerbating reward system tolerance (35).

Our study identified gender as a significant factor influencing the latent profiles of internet addiction among nursing students. Specifically, female nursing students exhibited a higher probability of membership in the moderate- and high- problematic internet use profile—a finding that diverges from prior literature (6). This discrepancy may be attributed to gender-based differences in coping styles: male students tend to internalize stressors or resort to gaming as an outlet (36), whereas female students are more inclined to seek emotional release through interpersonal communication. Our study identified physical activity as a significant factor associated with latent profiles of problematic internet use among nursing students. Consistent with prior findings, higher frequency of physical activity was associated with milder symptoms of internet addiction (37). Substantial intervention studies further confirm that physical activity can significantly alleviate internet addiction symptoms (38). Our study results indicate that nursing students from families with lower per capita monthly income are more likely to belong to the high- problematic internet use profile, which is consistent with previous research findings (39). This pattern may be attributed to greater sensitivity and lower self-esteem among students from economically disadvantaged families, which could further translate into increased reliance on the internet as a compensatory mechanism. Physical activity effectively reduces internet addiction levels, not only improving physical health but also enhancing self-esteem, willpower, and resistance to addictive online behaviors (40). While both internet use and physical activity provide interactive and recreational engagement, they differ in nature: immersive online participation detaches individuals into a virtual environment, whereas physical activity fosters real-world experiences by promoting physical and mental well-being (40). A key theoretical explanation is the time displacement effect, wherein physical activity occupies time otherwise spent online while subconsciously inducing positive physiological and psychological changes (41). Consequently, nursing students who regularly engage in physical activity tend to exhibit better overall health and healthier internet use patterns.

Network analysis aids in identifying central nodes within psychological structures, where nodes with high centrality exert a disproportionately strong influence on the entire network. Strategically targeting these core components in psychological interventions is meaningful, as changes in them can propagate to related peripheral symptoms, potentially reducing or eliminating the manifestation of co-occurring issues (42). Our centrality analysis of the problematic internet use network structure revealed variations in node connectivity across the four latent profiles. In the Low Problematic Internet Use Profile, RP-TM exhibited the highest strength centrality, indicating it was the most centrally connected node within this network. Furthermore, RP-IH showed the highest centrality in the Moderate Problematic Internet Use Profile, while Sym-C & Sym-W were most central in the High Problematic Internet Use Profile. Based on network theory, targeting highly central nodes could potentially influence adjacent symptoms and the broader network structure. Thus, developing profile-specific interventions tailored to these distinct connectivity patterns may offer a potential approach to addressing problematic internet use among undergraduate nursing students. Our findings provide a valuable, data-driven perspective for nursing educators in considering precise intervention content and formulating targeted strategies.

### Limitations

This study valuably applied network analysis to investigate problematic internet use among nursing students, performing LPA and subsequent network analysis. It offers a valuable perspective for gaining deeper insights into the symptom structure of problematic internet use in this population. However, several limitations should be acknowledged. First, network analysis has inherent methodological constraints, particularly its reliance on mathematical models and its sample-dependent nature. The identified network structure stems from our specific sample and may not generalize to different cultural contexts or healthcare systems. Second, regarding sampling, although a large sample was used, the convenience sampling approach may introduce selection and volunteer biases. Participants who volunteered might differ systematically from non-participants (e.g., in terms of internet usage habits). Additionally, the sample was drawn from specific institutions, which may not capture the diversity of nursing curricula and regional cultural factors. This homogeneity could affect the estimated proportions of latent profiles and the stability of network structures, limiting the generalizability of the findings to the broader nursing student population. Third, despite implementing quality control measures, reliance on self-reported data may introduce recall bias and social desirability bias, underscoring the need for multi-method assessments (e.g., clinical interviews, behavioral tracking) in future research. Finally, we recognize the limitations of the cross-sectional design in establishing causal relationships and plan to employ longitudinal designs in future studies to better understand temporal sequences and causal links among variables.

## Conclusion

In summary, this study identified four distinct profiles of problematic internet use among undergraduate nursing students, underscoring the potential value of precise interventions. Factors associated with higher-risk profiles included, lower monthly household income, and lower levels of physical activity. Furthermore, network analysis revealed variations in symptom connectivity patterns across these profiles. Consequently, our findings suggest that nursing educators may consider developing tailored intervention strategies that target the unique characteristics and symptom patterns of each identified profile.

## Data Availability

The original contributions presented in the study are included in the article/**Supplementary Material**. Further inquiries can be directed to the corresponding author.
