# Supplementary material for: Internet addiction among nursing students: application of latent profile analysis and network analysis
Source: Front Psychiatry. 2026 Apr 21;17:1814343. doi: 10.3389/fpsyt.2026.1814343 (PMC13139070; doi:10.3389/fpsyt.2026.1814343)
Supplement: Supplementary file 1 [file Supplementaryfile1.docx]

**Supplementary -1profile**

***Fig.1.Network structure……………………………………………………….. Page 2***

***Fig 2.*** ***Network Centrality Indices Plot*** ………………………………………... ***Page 2***

***Fig 3*. *Edge Weight Plot***………………………………………………………. ***Page 3***

***Fig 4. Stability coefficient of central indicators………………………………. Page 3***


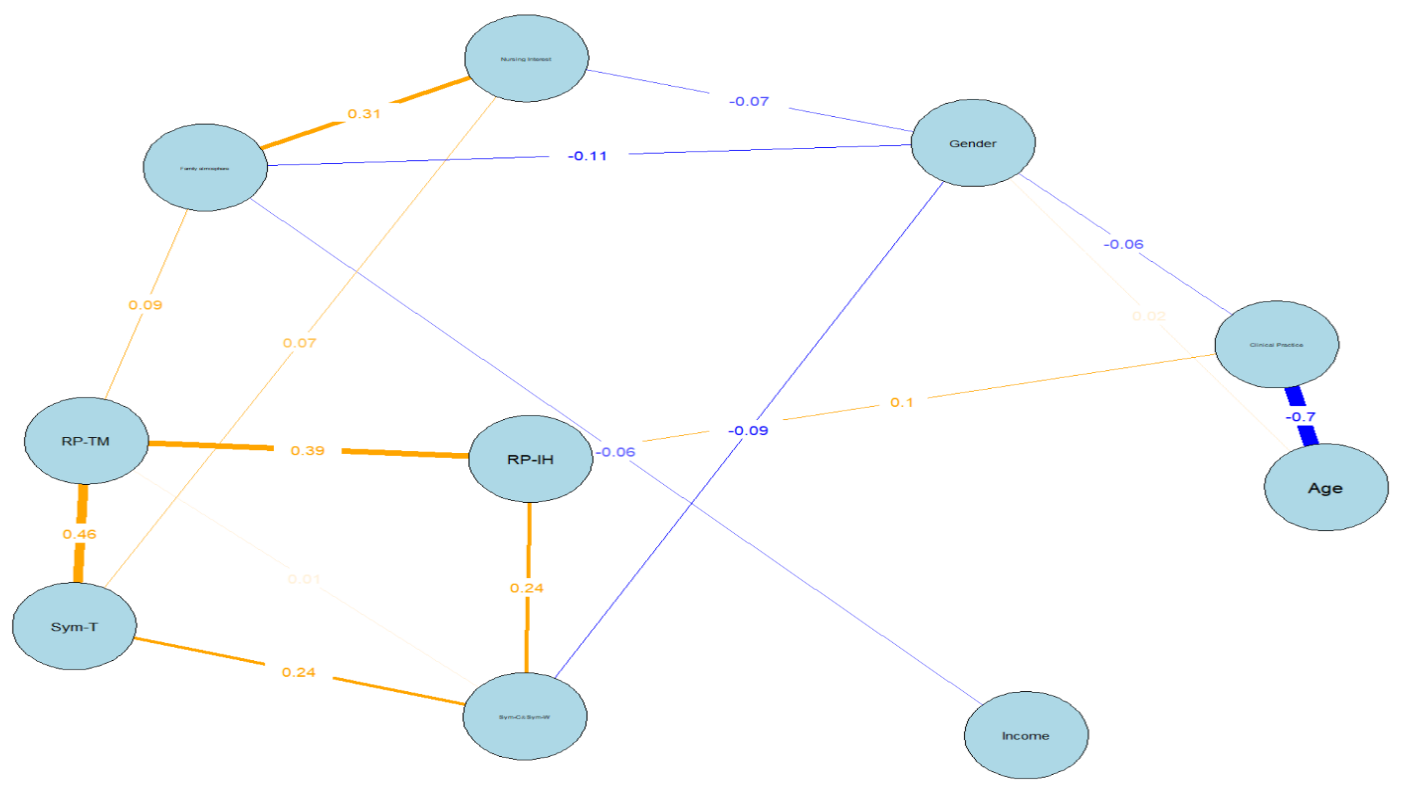


***Fig.1.Network structure***


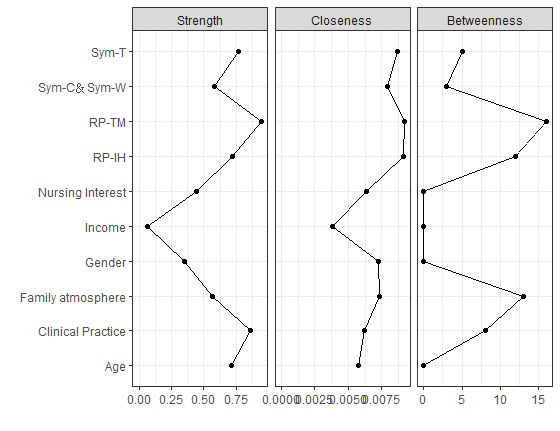


***Fig2. Network Centrality Indices Plot***


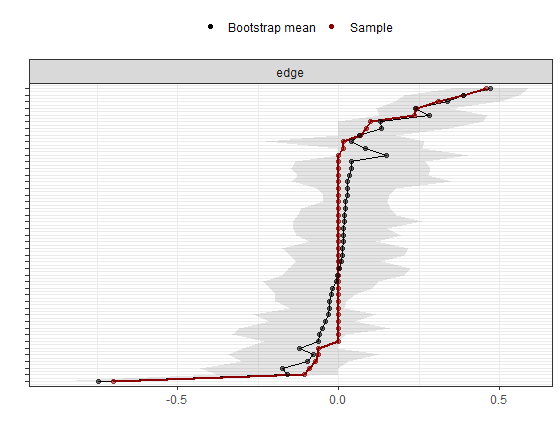


***Fig 3. Edge Weight Plot***


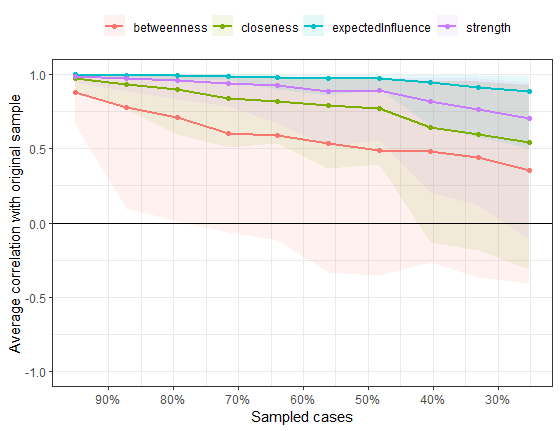
***Fig 4. Stability coefficient of central indicators***

**Supplementary -2profile**

***Fig 5. Network structure……………………………………………………….. Page 5***

***Fig 6.*** ***Network Centrality Indices Plot*** ………………………………………... ***Page 5***

***Fig 7*. *Edge Weight Plot***………………………………………………………. ***Page 6***

***Fig 8. Stability coefficient of central indicators………………………………. Page 6***


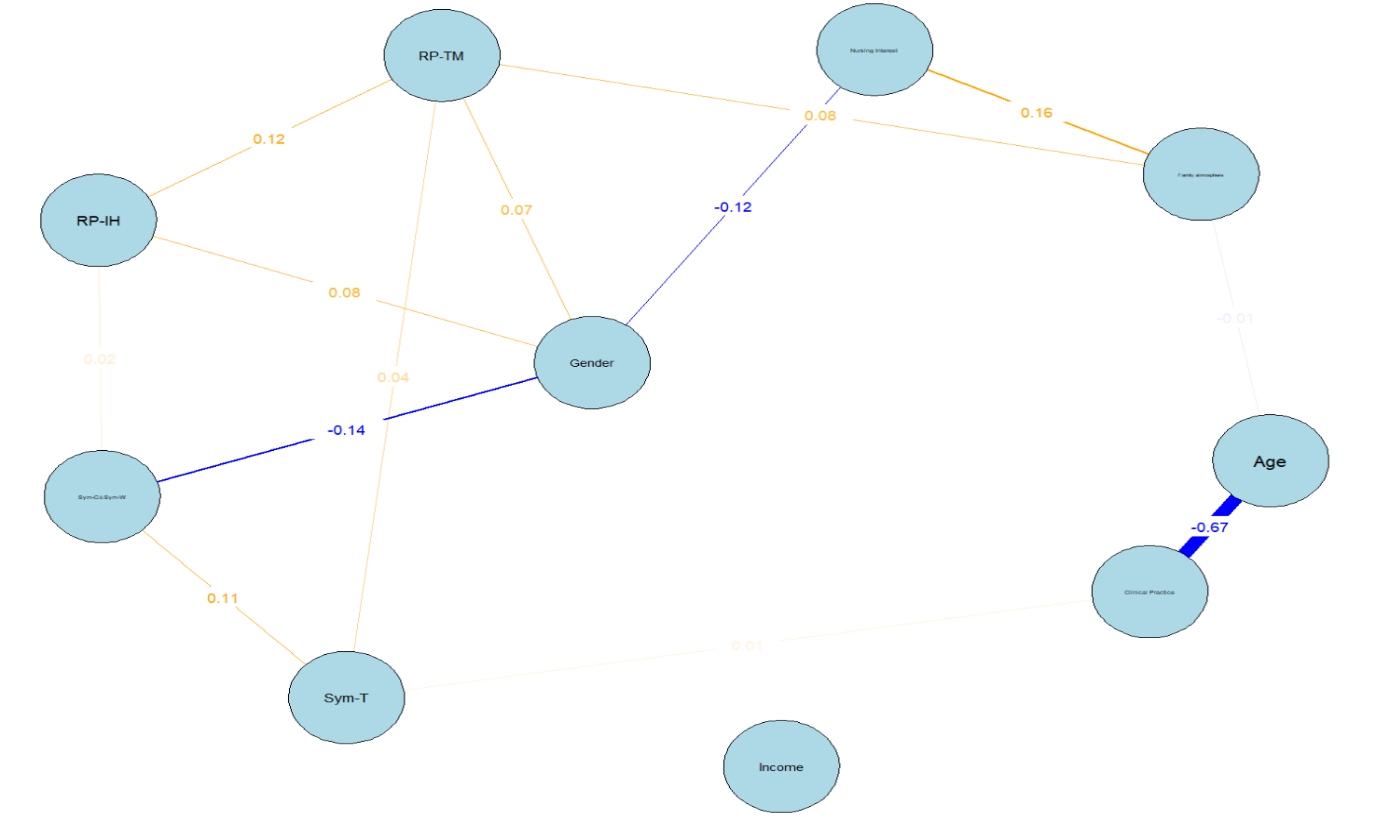


***Fig 5. Network structure***


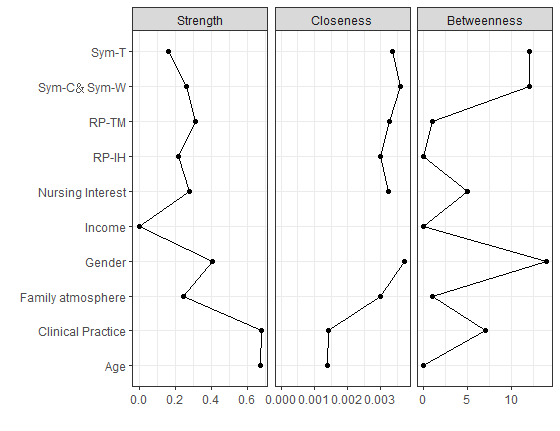


***Fig. 6. Network Centrality Indices Plot***


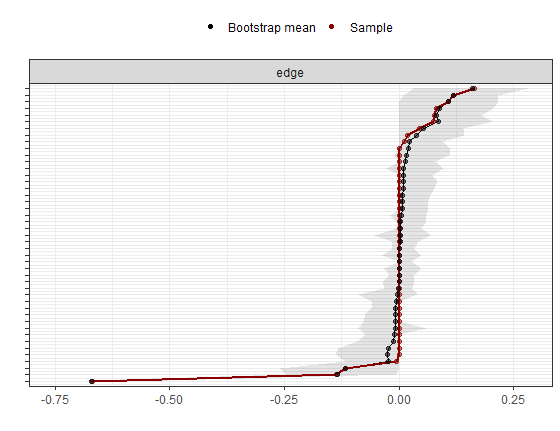


***Fig 7. Edge Weight Plot***


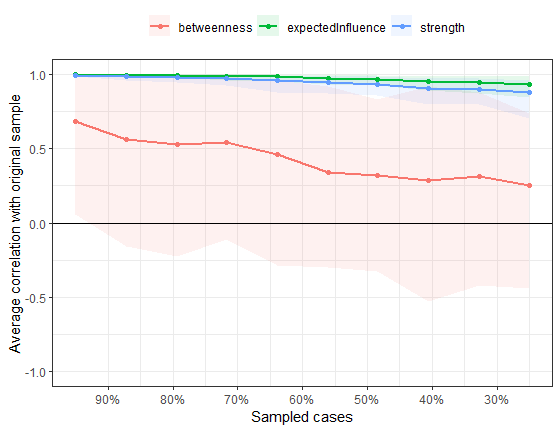


***Fig 8. Stability coefficient of central indicators***

**Supplementary -3profile**

***Fig 9. Network structure……………………………………………………….. Page 8***

***Fig 10.*** ***Network Centrality Indices Plot*** ……………………………………... ***Page 8***

***Fig 11*. *Edge Weight Plot***………………………………………………………. ***Page 9***

***Fig 12. Stability coefficient of central indicators……………………………… Page 9***


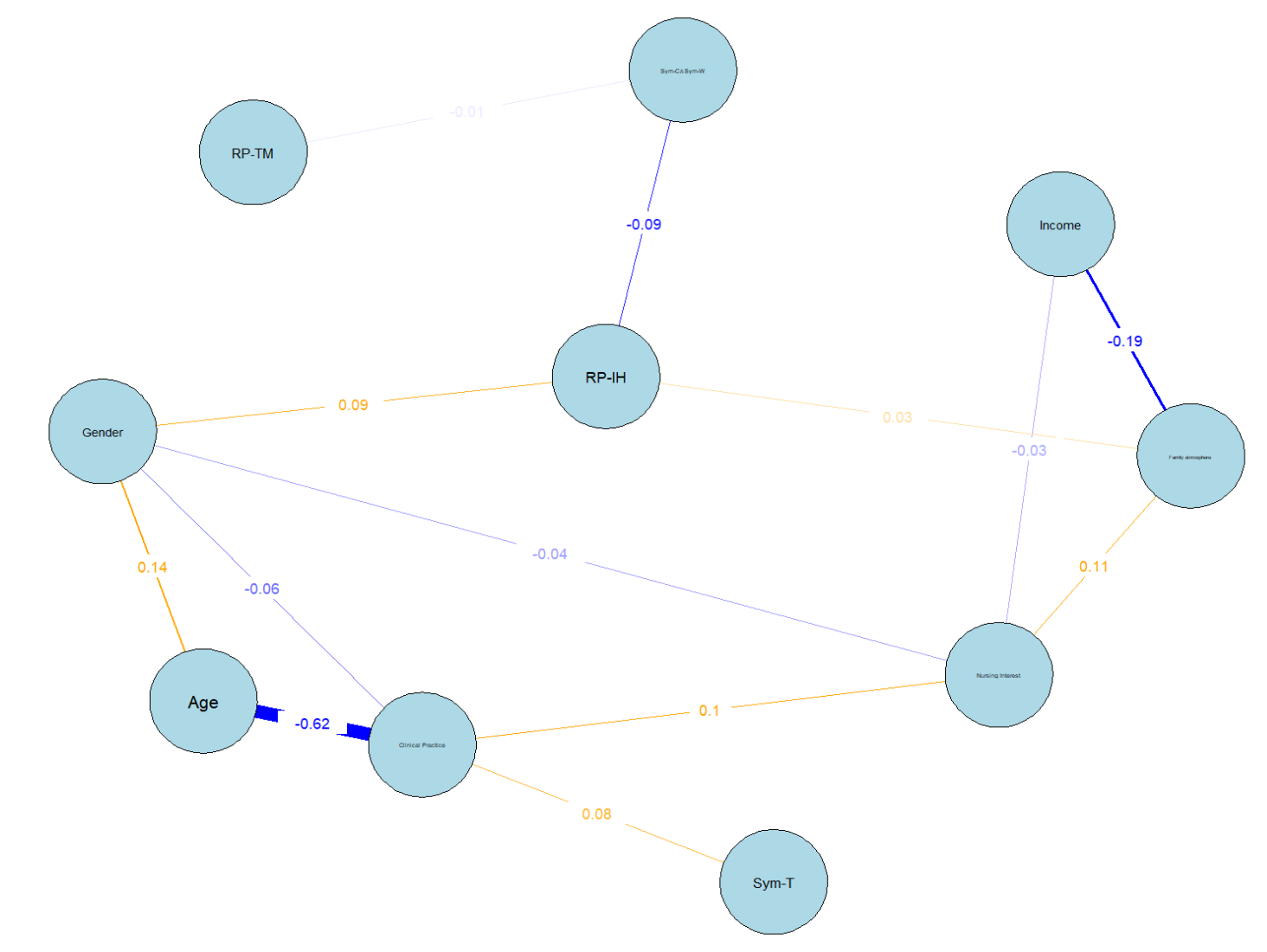


***Fig 9. Network structure***


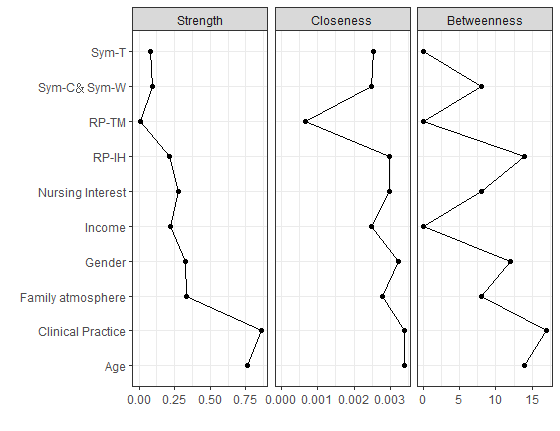


***Fig 10. Network Centrality Indices Plot***


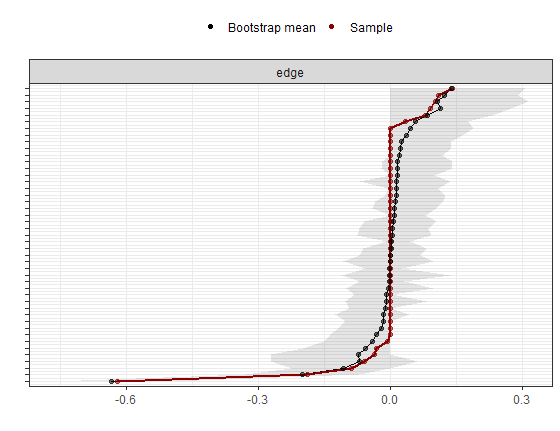


***Fig 11. Edge Weight Plot***


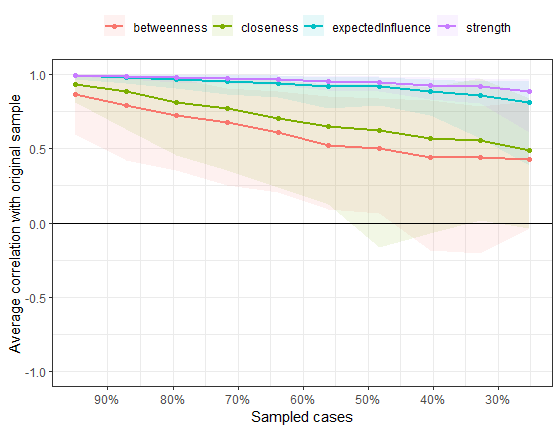


***Fig 12. Stability coefficient of central indicators***

**Supplementary -4profile**

***Fig 13. Network structure…………………………………………………… Page 10***

***Fig 14.*** ***Network Centrality Indices Plot*** ……………………………………... ***Page 10***

***Fig 15*. *Edge Weight Plot***……………………………………………………. ***..Page 11***

***Fig 16. Stability coefficient of central indicators…………………………… Page 11***


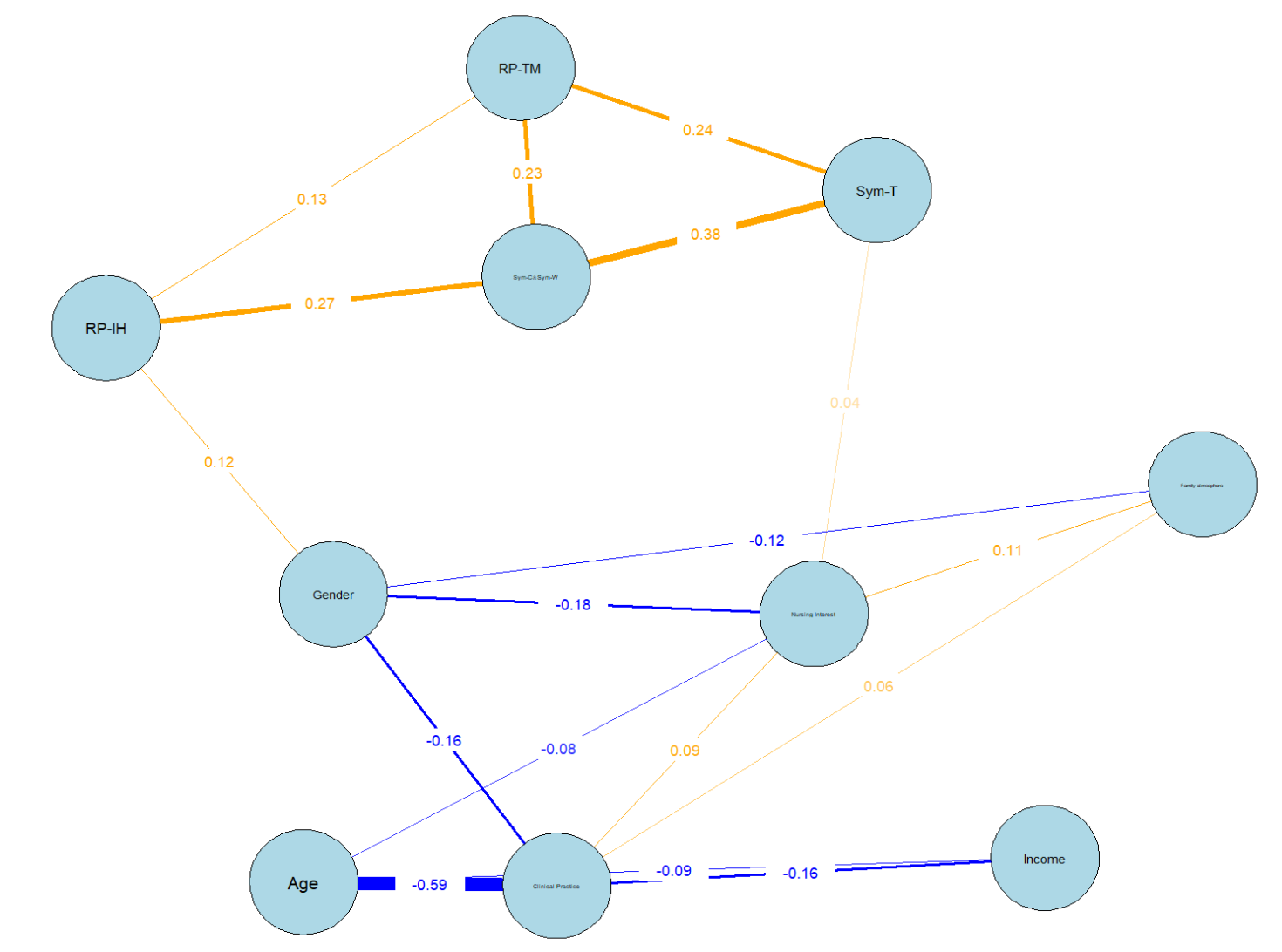


***Fig 13. Network structure***


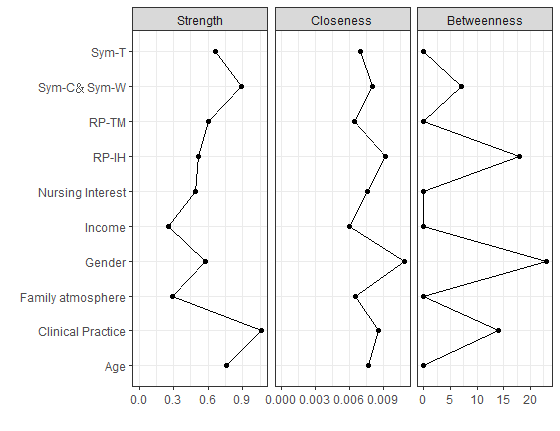


***Fig. 14. Network Centrality Indices Plot***


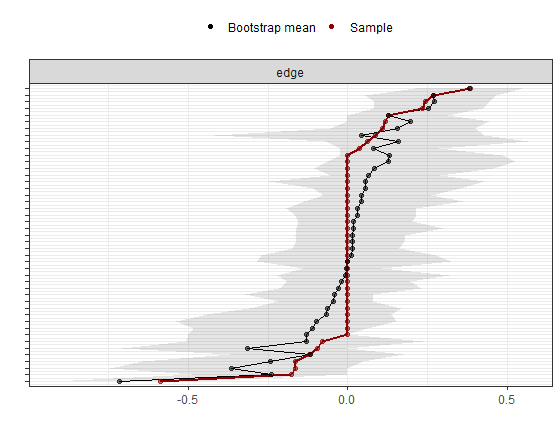


***Fig 15. Edge Weight Plot***


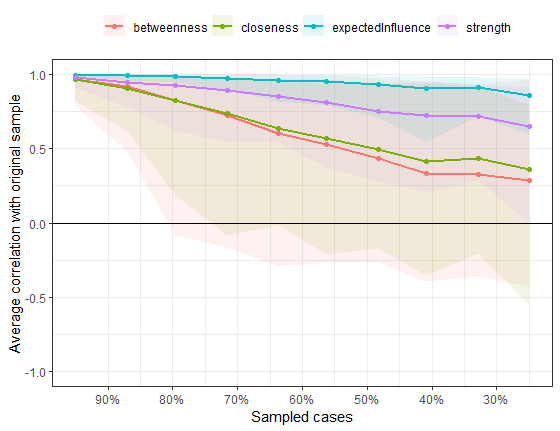


***Fig.16. Stability coefficient of central indicators***
